# Antarctic yeasts: potential use in a biologic treatment of textile azo dyes

**DOI:** 10.1186/s40643-022-00507-5

**Published:** 2022-03-09

**Authors:** F. Ruscasso, I. Cavello, G. Curutchet, S. Cavalitto

**Affiliations:** 1 Centro de Investigación Y Desarrollo en Fermentaciones Industriales (CINDEFI), UNLP, CCT La Plata-CONICET, Calle 47 y 115, 1900 La Plata, Provincia de Buenos Aires Argentina; 2 Instituto de Investigación E Ingeniería Ambiental -IIIA, UNSAM, CONICET, Campus Miguelete, 25 de mayo y Francia, 1650 San Martín, Provincia de Buenos Aires Argentina; 3 Escuela de Ciencia Y Tecnología E Instituto de Investigación E Ingeniería Ambiental, UNASM, CONICET, Av. 25 de Mayo y Francia, 1650 San Martín, Provincia de Buenos Aires Argentina

**Keywords:** *Leucosporidium muscorum* F20A, Cold-adapted yeasts, Textile wastewaters

## Abstract

We investigated the dye-removal potential of a collection of 61 cold-adapted yeasts from the King George Island, Antarctica, on agar plates supplemented with 100 mg L^–1^ of several textile dyes; among which isolates 81% decolorized Reactive Black 5 (RB-5), with 56% decolorizing Reactive Orange 16, but only 26% doing so with Reactive Blue 19 and Acid Blue 74. Furthermore, we evaluated the ligninolytic potential using 2,2ʹ-azino-bis(3-ethylbenzothiazoline-6-sulfonic-acid) diammonium salt-, 3,5-dimethoxy-4-hydroxybenzaldehydazine-, or manganese-supplemented plates but detected no activity, possibly due to a dye-removal mechanism involving reductases. The removal kinetics were studied in liquid medium supplemented with 100 mg L^–1^ of RB-5 in a selection of 9 yeasts. The highest volumetric-removal rates (η) were found for *Candida sake* 41E (4.14 mg L^–1^ h^–1^), *Leucosporidium muscorum* F20A (3.90 mg L^–1^ h^–1^), and *Cystofilobasidium infirmominiatum* F13E (3.90 mg L^–1^ h^–1^). Different UV–Vis spectra were obtained if the dye removal occurred by biodegradation or biosorption/bioaccumulation. *L. muscorum* F20A was selected to study the dye-removal mechanism of RB-5 and the effect of different chemical and environmental parameters on the process. Optimum dye-removal conditions were obtained with 10 g L^–1^ of glucose within an initial medium pH range of 5.0 to 6.0. Up to 700 mg L^–1^ of dye could be removed in 45 h. High-performance liquid chromatography profiles obtained were consistent with a biodegradation of the dye. Phytotoxicity was estimated by calculating the 50%-inhibition concentration (IC_50_) with *Lactuca sativa* L. seeds. These findings propose psychrophilic yeasts as a novel environmentally suitable alternative for the treatment of dye-industry wastewaters.

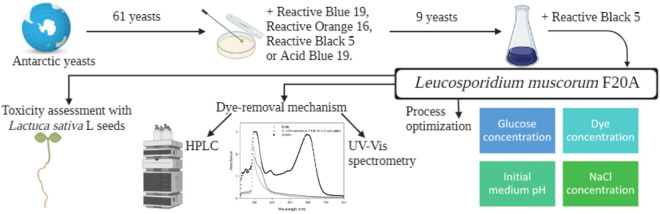

## Introduction

Textile industries consume a huge amount of water and generate large volumes of effluents containing a great variety of chemicals that are highly colored—such as dye, detergents, softeners, and other compounds of difficult degradation (Wang et al. 2011). These effluents exhibit elevated values of biochemical and chemical oxygen demands and tend to contain a high load of total dissolved solids plus total suspended solids and toxic compounds and a high pH and salinity (Katheresan et al. 2018). If textile wastewaters are discharged directly into water bodies, they can pollute the surrounding environment and cause problems by reducing light penetration, thus diminishing photosynthetic activity and the availability of oxygen that is essential for aquatic life forms. The dye molecules as well as the compounds derived from them—such as aromatic amines, metals, and chlorides—could be toxic to organisms found in the environment where those effluents are discharged (Tan et al. 2013; Jafari et al. 2014). In addition, mutagenic and carcinogenic effects in aquatic organisms by such wastewaters have been reported (Das and Charumathi 2012).

Dye concentration in textile effluents generally ranges from 10 to 200 mg L^–1^ (Jafari et al. 2013a). The textile dyes and their degradation products are considered xenobiotic compounds and can resist microbial and physicochemical attack. Hence, most dyes are not easily destroyed by conventional processes, thus constituting one of the most difficult types of effluent to effectively treat (Ramalho et al. 2002; Pajot et al. 2014). Biologic processes have been considered attractive because they are simple, inexpensive, and cause minimal environmental impact (Das and Charumathi 2012). Microbial dye-removal effectiveness depends on the adaptability and enzymatic activity of the microorganisms selected and on the dye's chemical structure (Yu and Wen 2005). By selecting the appropriate microorganism, dye wastewaters could be successfully treated by this approach.

Many microorganisms from different taxonomic groups have been found capable of removing dyes from textile effluents: these taxa include bacteria, filamentous fungi, ascomycetous and basidiomycetous yeasts, and algae (Yu and Wen 2005; Wang et al. 2009; Enayatizamir et al. 2011; El Bouraie and El Din 2016). Biologic dye removal generally occurs through three mechanisms: bioadsorption, bioaccumulation, and biodegradation. Yeasts have many advantages over filamentous fungi and bacteria, such as growing as fast as bacteria along with their fungal ability to resist unfavorable environments. Certain yeasts have been reported to be very efficient in treating high-resistance organic wastewater (Das and Charumathi 2012). Thus far, several ascomycetous yeast species have been reported to be capable of treating dye-containing wastewaters—such as *Candida albicans, C. boidinii, C. krusei, C. oleophila, C. tropicalis, C. zeylanoides, Cyberlindnera saturnus, Debaryomyces polymorphus, Galactomyces geotrichum*, *Issatchenkia occidentalis, and Pichia occidentalis* (Jafari et al. 2013b; Sampaio et al. 2018). Among the basidiomycetous *Cryptococcus heveanensis* (bioadsorption), *Rhodotorula minuta* (bioaccumulation), *Pseudozyma rugulosa and Sterigmatomyces halophilus* (biodegradation), and species of the genus *Trichosporon: T. akiyoshidainum, T. beigelli, T. multisporum, T. porosum* (biodegradation) have likewise been reported with the same capability (Pajot et al. 2014; Al-Tohamy et al. 2020).

The enzymes of cold-adapted microorganisms have been studied especially in recent years because of potential application of those taxa in biotechnologic processes, but only a few reports have treated the application of psychrophilic microbes in bioremediation. Moreover, among those microorganisms studied, yeasts have notably constituted a considerably small fraction. Some investigations involving Antarctic yeasts and their use in bioremediation are mentioned in a book chapter by Vero et al. (2021). The application of Antarctic yeasts specifically in the treatment of textile wastewater has been scantily investigated; To the best of our knowledge, it was only studied by Rovati et al. (2013) and in our own previous investigations (Ruscasso et al. 2021a, b), on solid media and in liquid media, respectively. The main objective of this study was therefore to identify species among a collection of Antarctic yeasts that would be capable of removing different dyes and then to investigate their potential application in textile-wastewater treatment. Psychrophilic or psychrotolerant yeasts that can remove dyes at room temperature are attractive from a technologic and operational point of view, since no energy or temperature-controlled premises would be required to heat the culture, thus reducing the operational costs of such an effluent treatment.

## Materials and methods

### Microorganisms and dyes

The study involved a collection of 61 yeasts from different samples of terrestrial and marine waters and soil from the King George Island, Antarctica. The yeasts were isolated, grown in Yeast Extract Peptone Dextrose (YPD) medium, and identified by Martinez et al. (2016). The specimens were freeze-dried and cryopreserved in the Microbiological Culture Collection of the CINDEFI-CONICET Institute, La Plata, Argentina and in the UdeLar Culture Collection of the Cátedra de Microbiología, Facultad de Química, Montevideo, Uruguay.

The dyes Reactive Black 5 (RB-5), Reactive Orange 16 (RO-16), Reactive Blue 19 (RB-19), and Acid Blue 74 (AB-74) were kindly supplied by a local company (ALCONIC SRL, Buenos Aires, Argentina). Table 1 presents some physicochemical characteristics of these dyes, such as the maximum wavelengths, the dye types, and the molecular weights. A stock solution of each one was prepared, filter-sterilized, and then stored at 5 ± 2 °C until use. Because RB-5 is one of the most commonly used in this industrial sector (El Bouraie and El Din 2016), we selected that compound as a model dye for all the assays in liquid medium.

**Table 1:** Dye physicochemical characteristics

Dye	Type of dye	Maximum wavelength (λ in nm)	Molecular weight (g mol^–1^)
Reactive Black 5	Double azo	595	991.82
Reactive Orange 16	Single azo	494	617.50
Reactive Blue 19	Anthraquinone	595	626.55
Acid Blue 74	Indigoid	609	466.35

### Screening for dye removal on solid media

The yeasts were screened for their color-removal capability on agar plates containing Normal Decolorization Medium (NDM; (Ramalho et al. 2002) supplemented with 100 mg L^–1^ of dye. As negative control, a plate for each dye was left without inoculation to confirm that dye decolorization was not due to abiotic conditions or environmental parameters. As positive (biotic) control, plates without dye were also inoculated. All the plates were incubated at 20 ± 2 °C and observed daily. The dye decolorization of the medium was examined visually by the disappearance of color around the colony, and the color acquired by the yeast biomass after dye removal was observed. This latter parameter was taken as a qualitative indicator the dye-removal mechanism involved (Das and Charumathi 2012).

### Oxidative-enzyme screening on agar plates

The ligninolytic potential was evaluated through the use of ABTS- ((2,2ʹ-azino-bis(3-ethylbenzothiazoline-6-sulfonic acid) diammonium salt, Sigma-Aldrich, Germany), syringaldazine- (SYR, 3,5-dimethoxy-4-hydroxybenzaldehydazine, Sigma-Aldrich, Germany), or manganese- (MnCl_2_.4H_2_O, Mallinckrodt Chemical, USA) supplemented plates (Steffen et al. 2000). The yeast strains were precultured on YPD-agar and then inoculated onto NDM-agar supplemented with 100 mg L^–1^ of ABTS, SYR or Mn^2+^. Solutions of ABTS, SYR, and Mn^2+^ were filter-sterilized and then added aseptically to NMD-agar medium.

The plates were examined daily by visual inspection of their degree of coloration. The presence of extracellular oxidative enzymes on the ABTS-plates was recognized by the generation of either a dark-green zone around colonies, corresponding to the radical-containing monovalent cation ABTS^+^ (blue-green coloration) or the divalent ABTS^2+^ (purple coloration). SYR-plates were observed for pink-purple coloration corresponding to the oxidized syringaldazine, indicating laccase activity. Plates containing Mn^2+^ were evaluated for the appearance of black or dark brown coloration corresponding to manganese oxide (II), that compound being produced by the action of peroxidase enzymes. In addition, to analyze whether the enzymes tested were inducible, the effect of preincubation with dyes and/or Mn^+2^ was evaluated. A positive control was performed with the white-rot fungus *Trametes versicolor*.

### Kinetics of dye removal

The dye-removal kinetics were studied in 250-ml Erlenmeyer flasks containing 50 ml of NDM liquid medium supplemented with 100 mg L^–1^ of RB-5. The flasks were inoculated with the strains that displayed haloes of decolorization on solid media upon an overnight incubation (optical density, OD_600_ = 0.06), at 20 ± 2 °C with rotary agitation (150 rpm) up to a total decolorization of the medium. Controls without dye or without yeast were also performed. At predetermined time intervals, samples were withdrawn and centrifuged at 4000×*g* for 10 min and the medium pH, OD_600_, biomass concentration, and dye removal monitored. The percent dye removal was calculated according to the following equation [Eq. (1)] from the measurement in a spectrophotometer (Beckman Coulter Inc., United States) of supernatant absorbance at 595 nm (*λ*), the RB-5 maximum wavelength (Rosu et al. 2018):

1 $${\text{dye removal}} = \left[ {\left( {A_{0} - A} \right)/A_{0} } \right] \times {1}00,$$

where *A*_0_ and *A* are the absorbance at the start and at time *t*, respectively. In addition, the yeast growth was assessed by OD_600_ and yeast biomass concentration (g L^–1^) by the gravimetric method (Lucas et al. 2006). Finally, glucose depletion was determined by the glucose-oxidase–peroxidase method (Glycemia, Weiner Lab., Argentina).

In addition, it was calculated the average specific removal rate (ν), the relationship between the amount of dye removed (mg L^–1^) per gram of biomass per time, and the average volumetric-removal rate (*η*): the amount of dye removed per liter per time.

### Dye-removal analysis

To study the possible RB-5 dye-removal mechanism by *Leucosporidium muscorum* F20A, samples of dye-containing cultures were withdrawn at predetermined time intervals, centrifuged for 10 min at 4000×*g* and the supernatants filtered through a 0.45-µm cellulose membrane (Sartorius, Germany) followed by analysis by UV–Vis spectrometry and high-performance liquid chromatography (HLPC).

UV–Vis spectra were performed spectrophotometrically (Beckman Coulter Inc., United States) to evaluate the appearance of new absorbance peaks corresponding to the production of aromatic amines by dye degradation and to observe the decrease in the dye-associated peak at the maximal absorbance in the visible range (Pereira et al. 2013). In addition, reversed-phase HPLC was performed. The metabolites were extracted from the supernatants with equal volumes of ethyl acetate (Cicarelli Lab., Argentina). The extracted organic phases were left to evaporate to dryness and then dissolved in 1.0 ml of absolute methanol (Sigma-Aldrich, Germany; (Ruscasso et al. 2021b). The resulting solutions were analyzed by a Waters HPLC system (pump model 510, Waters Corporation, USA) on an XBridge C18 column (particle size, 5 µm; I.D., 5.6 × 250 mm). The mobile phase was acetic acid plus nanopure water, pH = 4.0 plus methanol and was delivered at a flow rate of 1.0 mL min^–1^ in gradient elution. The samples were analyzed with a diode-array detector at 260 nm by means of Empire Pro software. The injection volume was 20 µL.

### Effect of different parameters on dye removal

As a first step to optimize the process, we studied the effect of different chemical and culture parameters on dye removal. Erlenmeyer-scale cultures of *L. muscorum* strain F20A were used under the same conditions as described previously, and each assay was carried out in duplicate. Biotic controls (without dye) were also performed. The effect on dye-removal efficiency of the glucose concentration (0–20 g L^–1^), the dye concentration (100–700 mg L^–1^), the initial medium pH (4.0–8.0), and the sodium chloride concentration (0–50 g L^–1^) were studied. The biomass concentration and the percent dye removal were analyzed and expressed as the means ± SD.

### Toxicity assessment with seeds from *Lactuca sativa* L.

To evaluate the phytotoxic response, *L. sativa* L. seeds were used as bioindicators of the level of toxicity. The tests were performed in quadruplicate on a set of dilutions (10, 25, 50, and 100%) with samples that were untreated or treated for 24, 48, 72, and 144 h. A negative control with distilled water and a positive control with a ZnSO_4_ were used. Twenty seeds per dilution were incubated in Petri dishes at 25 °C ± 2 °C for 5 days in the dark (Sobrero and Ronco 2004). After 5 days, the germinated seeds were harvested, and the root and hypocotyl elongation measured. The toxicity was estimated by calculating the concentration producing a 50% inhibition (IC_50_) (IRAM 2008).

## Results and discussion

### Dye-removal screening on solid media

In order to select the yeasts with potential application in the treatment of dyes in wastewater, we first performed a solid-medium screening. Agar plates were observed daily to determine colony color and detect the presence of a colorless halo. Colony color was considered a qualitative parameter of what dye-removal mechanism could be occurring: an uptake of the dye color by the colony could indicate either bioaccumulation or bioadsorption; whereas, a colony maintaining its original color would be performing a biodegradation. After 72 h, 35 yeasts produced a halo of decolorization on the solid media supplemented with 100 mg L^–1^ of RO-16, with 39% maintaining the original colony color. In addition, 50 yeasts produced a halo of decolorization on the RB-5 plates, with 37% retaining the original color. This last observation would indicate that the mechanism involved in the dye removal could be biodegradation rather than adsorption or accumulation by the yeasts. Only 16 yeasts were able to remove Reactive Blue 19 and Acid Blue 74. Table 2 provides the experimental data obtained from the dye-removal screening on solid medium. Of all the yeasts studied, only five were able to remove 3 of the 4 dyes assayed and also maintain the original colony color—those pertaining to the genera *Candida*, *Debaryomyces*, and *Leucosporidium*—whereas 12 of the yeasts studied eliminated 2 of the 4 dyes assayed while retaining colony color and were members of the genera *Rhodotorula*, *Candida*, and *Debaryomyces*. To the best of our knowledge, to date only two surveys related to Antarctic yeasts and their potential in dye removal have been reported: The first was conducted by Rovati et al. (2013), who found that 33 percent of 61 yeasts isolated from King George Island were capable of removing a mixture of four dyes in solid medium at a final concentration of 200 mg L^–1^ between 10 to 20 days of culture at 15 °C. The second was performed by Bezus et al. (2021) who reported that 32 psychrophilic and psychrotolerant yeast isolates were able to remove at least one of the dyes used (RO-16, RB-5, and RB-19).

**Table 2:** Comparison through the use of different dyes of the percentages of cold-adapted yeasts producing a decolorization halo and maintaining the original color of the colony

Dyes	Production of decolorization halo (%)	Maintenance of colony color (%)
Reactive Black 5	81	37
Reactive Blue 19	26	5
Reactive Orange 16	56	39
Acid Blue 74	26	23

There are other studies that reported the ability to remove dyes by yeast, for example Sampaio et al. (2018) isolated 92 yeasts from Mediterranean forested wetland, of which 43 were able to remove at least one dye essay, the basidiomycetous yeasts *Tremellales, Sporidiobolales* and *Filobasidiales* were the ones that presented the greatest potential for the removal. On the other hand, Pajot et al. (2007) reported that 37% of the 63 yeasts isolated from Las Yungas rainforest showed the highest removal rate of each of the dyes tested. In the research from Dias et al. (2010), 92 yeast were isolated from decomposing leaves in a freshwater marsh, 12 of them showed good dye-removal abilities, corresponding to 7 species: only one ascomycetous (*Candida parapsilosis*) and six basidiomycetous (*Filobasidium sp., Rhodosporidium kratochvilovae, Rhodotorula graminis, Cryptococcus laurentii, C. podzolicus, and C. victoriae)*.

### Oxidative-enzyme screening on agar plates

Dye biodegradation by yeasts can occur through reductive or oxidative enzymatic reactions. Ligninolytic enzymes are involved in dye oxidation, including laccase, manganese peroxidase, tyrosinase, and lignin-peroxidase (Martorell et al. 2017). Preliminary assays were carried out to evaluate the participation of oxidative enzymes in the yeast isolates. We found no evidence that any of those specimens could produce a halo of enzymatic activity on solid media (after 4 weeks of incubation)—and even after preincubation with RB-5 or manganese (II). Therefore, those yeasts very likely did not have inducible oxidative enzymes. A possible explanation for those results may be that in those yeasts where a positive decolorization was observed on the dye-containing agar plates the enzymes involved could be reductases. In this regard, Šlosarčíková et al. (2020) reported that the first degradation step—the cleavage of the azo bond (–N=N–)—could be catalyzed by reductases, such as azoreductase or NADH-dichlorophenolindophenol reductase. Further studies, however, need to be carried out in order to evaluate the possible participation of such reductases.

The azo dyes in this study were more easily removed than RB-19 and AB-74. This difference could be attributed to the nature of the dyes and the absence of oxidase enzymes. This finding was contrary to previous studies that had suggested that anthraquinone dyes and indigo carmine (AB-74) were more easily removed than azo dyes (with one or two azo bonds) because of the former two's chemical structure along with the presence of oxidase enzymes (Abadulla et al. 2000; Pajot et al. 2007).

### Kinetics of dye removal

Batch cultures were conducted to study the dye-removal kinetics in NDM liquid medium supplemented with 100 mg L^–1^ of dye. RB-5—commonly used in the dyeing of cotton, cellulose fibers, wool, and nylon—was used as a model dye for all the liquid-medium experiments in this work. A selection of 9 cold-adapted yeasts was chosen, with 5 being selected for their ability to remove 3 of the 4 dyes tested. Next, among the 12 yeasts capable of producing a halo of decolorization in solid medium with 2 of the 4 dyes studied, the one that was different in genus and species was chosen. Finally, 3 yeasts were randomly selected among those capable of producing a halo only on the medium supplemented with RB-5. Table 3 lists the genus and species of each selected yeast and the results obtained in the dye-removal study. Among the reported yeasts with the greatest potential to remove dyes are the genera *Saccharomyces, Trichosporon, Debaryomyces, Candida, Cryptococcus, Pichia, Kluyveromyces* and *Rhodotorula* (Sampaio et al. 2018; Al-Tohamy et al. 2022).

**Table 3:** Genus and species of the yeasts selected for the study of dye-removal kinetics and the kinetic parameters of percent color removal and biomass concentration after 24 h

Yeast	Phylogenetic affiliation	Access number	Dye removal (%)	Biomass concentration (g l^–1^)	Specific dye-removal rate (mg g^–1^ h^–1^)	Volumetric dye-removal rate (mg L^–1^ h^–1^)
12R	*Candida glaebosa*	KU659493	35.6	1.99	0.74	1.48
12D	*Candida glaebosa*	KU659494	44.5	3.50	0.53	1.85
41E	*Candida sake*	KU659517	99.5	5.05	0.82	4.14
F13E	*Cystofilobasidium infirmominiatum*	KU659531	93.6	3.98	0.98	3.90
F9D	*Debaryomyces hansenii*	KU659525	35.7	2.67	0.56	1.49
F12B	*Debaryomyces hansenii*	KU659527	14.2	2.03	0.29	0.59
F39A	*Debaryomyces hansenii*	KU659542	13.7	2.07	0.28	0.57
F20A	*Leucosporidium muscorum*	KU659533	93.5	4.36	0.89	3.90
F12D	*Rhodotorula laryngis*	KU659529	29.1	1.00	1.21	1.21

What is noteworthy is that no reduction in dye concentration was observed in any of the abiotic controls: therefore, the decolorization of the inoculated media could be attributed to the yeast activity. All the yeasts selected were able to decolorize the medium supplemented with 100 mg L^–1^ of the RB-5 dye. Moreover, the most pronounced reduction in dye concentration occurred during the exponential growth phase, which stage was associated with the primary metabolism of the yeast. By contrast, in filamentous fungi, azo-dye removal occurs during secondary metabolism and requires longer incubation time. This finding was also reported by Lucas et al. (2006) and Jafari et al. (2013b). At 12 h of culture time, we observed that the biomass concentration was proportional to the percent RB-5 removal, with three of the yeast isolates removing more than 90% of the RB-5 at this time—namely, *Candida sake* 41E (5.05 g biomass L^–1^), *Cystofilobasidium infirmominiatum* F13E (3.98 g biomass L^–1^), and *L. muscorum* F20A (4.36 biomass L^–1^).

The average specific removal rate (*ν*) and the average volumetric-removal rate (*η*) were calculated (cf. Table 3). The first parameter proved to be the highest for the isolate *Rhodotorula laryngis* F12D (1.212 mg RB-5 cell^–1^ h^–1^) followed by *C. infirmominiatum* F13E and *L. muscorum* F20A (0.979 mg RB-5 cell^–1^ h^–1^ and 0.893 mg RB-5 cell^–1^ h^–1^, respectively). Similar values had been previously reported for the dye degradation of several yeasts and for dye-accumulating ascomycetous yeasts including *Kluyveromyces marxianus*, *C. tropicalis*, and *Saccharomyces cerevisiae* (Pajot et al. 2007*).* Although we should note that, in some references cited, higher dye concentrations or dyes bearing simpler chemical structures had been used. *Rhodotorula* sp. corresponds to one of the first yeast biomass reported to remove dyes, whereas Rovati et al. (2013) reported an *Rhodotorula laryngis* with dye removal potential. For the parameter η the highest values recorded occurred for the yeasts *C. sake* 41E (4.14 mg L^–1^ h^–1^), *L. muscorum* F20A (3.90 mg L^–1^ h^–1^) and *C. infirmominiatum* F13E (3.90 mg L^–1^ h^–1^). When we initially proposed biodegradation as a dye-removal mechanism, from observing the biomass color, we found the dye-removal kinetic parameters to be higher than the values obtained upon proposal of bioaccumulation.

The liquid samples were centrifuged and the pellets washed three times, after which step the only biomasses that had taken up the color of the dye corresponded to the *Debaryomyces* species (*n* = 3). Thus, their dye-removal mechanisms were dye bioadsorption or bioaccumulation, whereas the mechanism of dye removal in the other six yeasts could be attributed to biodegradation.

**Fig. 1:**
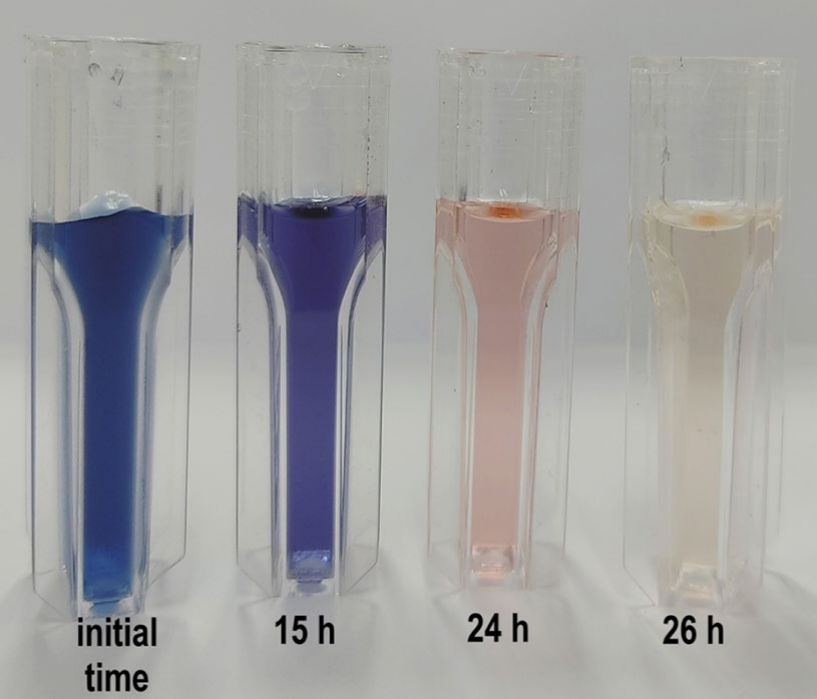
Different stages of RB-5 removal (100 mg L^–1^) by *Candida sake* 41E. The figure depicts the sequential shift in the color of the liquid samples upon exposure to the psychrophilic yeast from the starting hue of deep blue through a violet at 15 h to a very light pink at 24 h to finally reach a completely colorless state after a further two hours

In the yeasts where a biodegradation mechanism was proposed, the removal occurred in stages. First, the shift from deep blue to violet was observed, then the cultures took on a pink coloration, and finally the color approached that of the biotic control (Fig. 1). This mechanism in stages agrees with evidence from previous observations for *K. marxianus, C. oleophila, C. boidinii* yeasts isolated from different environment of the earth (Meehan et al. 2000; Lucas et al. 2006; Martorell et al. 2016).

**Fig. 2:**
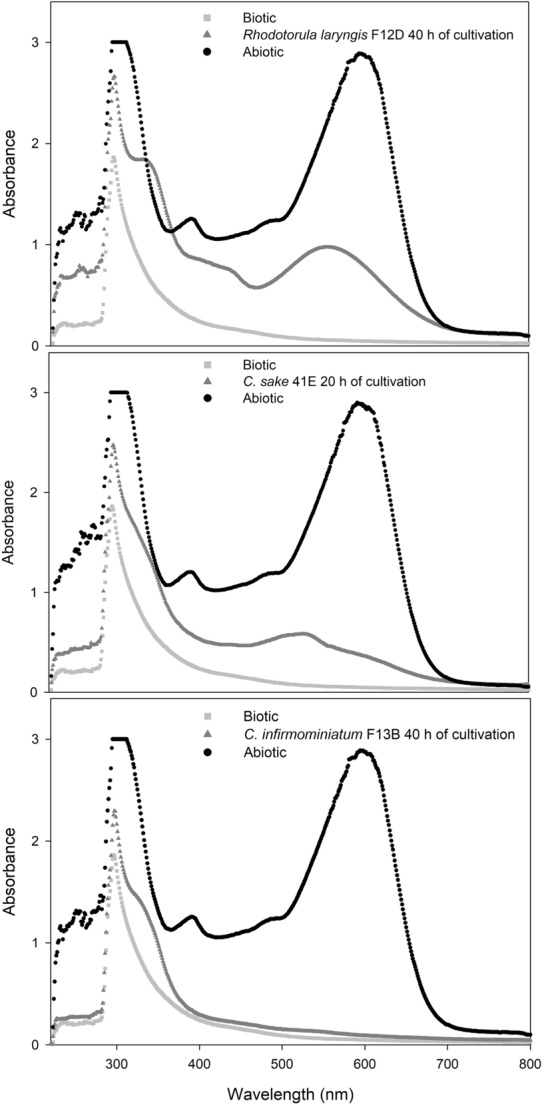
UV–visible scans from batch cultures of *Rhodotorula laryngis*, *Candida sake*, and *Cystofilobasidium infirmominiatum* containing 100 mg L^–1^ of Reactive Black 5 at different incubation times. The three spectra illustrate the decrease in absorbance of the dye obtained from the starting abiotic control (situation without yeast, black line, top record) to that occurring after incubation with the yeast (dark-gray line, middle record), compared to the spectrum of the biotic control (situation in the absence of dye, light-gray line, bottom record)

The analysis of the absorption spectrum of the supernatant differs in each dye-removal mechanism. In bioadsorption, the absorption spectrum displays all the peaks decreasing approximately in proportion to each other. In contrast, if the biodegradation of the dye occurs, the main absorbance peak in the visible region would gradually decrease until disappearance; and when the azo dye degrades to colorless aromatic compounds, those compounds could develop new absorbance peaks (Isik and Sponza, 2003; Das and Charumathi, 2012). A UV–visible analytical scan (200–800 nm) of the untreated dye solution containing RB-5 presents two main absorption peaks at 595 nm and 310 nm, attributed to the presence of an azo-bond chromophore and to aryl or naphthalene rings, respectively (Feng et al. 2000; Wang et al. 2014). Figure 2 illustrates the spectral scan of selected supernatants of the simulated effluent after yeast treatment. With increased culture time, the peak in the visible region decreased compared to the peak height in the abiotic control, while a displacement of the peak at 310 nm occurred, corresponding to the generation of aromatic amines. These features indicate the breakdown of the azo groups that were not completely mineralized. Whereas a complete removal of color in the medium (absorbance at 595 nm) occurred within 24–48 h, the degradation of aromatic–dye–degradation residues (absorbance at 310 nm) appeared to be more recalcitrant. An increase in the peak intensity at 254 nm in the UV region was observed, that represented benzene groups (Ong et al. 2012). This increase would indicate the accumulation of aromatic amines throughout the process.

### Dye-removal analysis

The Antarctic yeast *L. muscorum* F20A strain was selected to study the degradation of the RB-5 azo dye through an analysis of the mechanism carried out along with the influence of different conditions on decomposition efficiency.

We selected the yeasts that produced a halo of decolorization on solid medium and maintained the colony coloration. Among those, *L. muscorum* F20A was chosen for the studies because that species was capable of completely decolorizing the medium in fewer than 48 h. Moreover, the yeast-biomass coloration was not altered, indicating the absence of any adsorption phenomenon and suggesting that biodegradation mechanisms were occurring in the dye removal (Ramalho et al. 2002). No variation in color intensity was observed in the negative (abiotic) control, thus suggesting that no external agency was affecting the process. *Leucosporidium muscorum* is a psychrotolerant yeast that belongs to the order Leucosporidiales (de García et al. 2015) and has been isolated from water samples from glaciers in Norway, from Argentina, from the Arctic Archipelago (Buzzini et al. 2012), and from the intertidal zone as well as from soil samples from Antarctica (Martinez et al. 2016; Freire et al. 2020). Despite the biotechnologic significance of cold-adapted microorganisms, up to the present time only a very limited number of reports related to the application of these psychrotolerant taxa in the treatment of different types of pollutants have appeared in the literature. With regard to *Leucosporidium,* only Csutak et al. (2010) reported its application in bioremediation and discussed its biodegradation properties.

In the present study, *L. muscorum* F20A could not use RB-5 as a sole carbon source for cell growth; rather, dye removal depended on an added source of carbon. This observation implies a cometabolic pathway for the degradation of this compound. A similar finding was also reported for the removal of several azo dyes by other yeast strains (Yang et al. 2008; Jafari et al. 2013b). As was mentioned above, the first azo-dye degradation step could result from the action of reductase enzymes; therefore, the assimilation of a carbon source and its subsequent metabolism would provide the yeast cells with the reducing power (NADH and or FADH_2_) required for that reaction.

In order to confirm the degradation mechanism of RB-5, UV–Vis–spectrum and HPLC analyses were performed for cultures supplemented with 200 mg L^–1^ of dye. The UV–Vis spectrum results were consistent with the occurrence of a partial mineralization of the metabolites generated from RB-5 degradation by *L. muscorum* F20A. This partial mineralization had been previously reported for aerobic biologic processes involving activated sludge (Bonakdarpour et al. 2011; Vyrides et al. 2014).

**Fig. 3:**
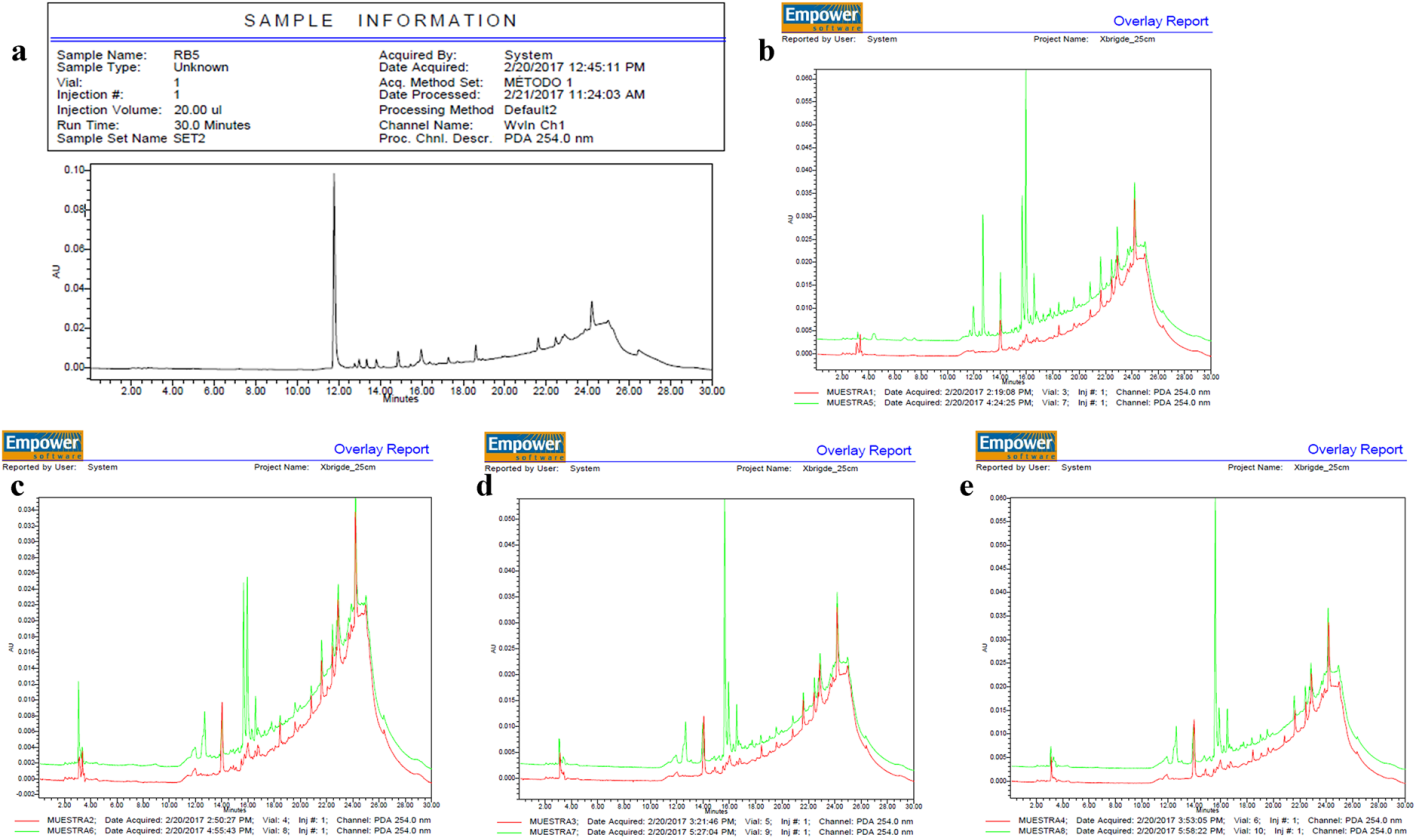
Chromatograms of the ethyl acetate extracts of supernatants at the final time point in the culture of *Leucosporidium muscorum* F20A supplemented with 200 mg L^–1^ of RB-5. The sample number and color code, the date, and other information pertaining to the run is provided below each chromatogram. For an explanation of the significance of the various peaks illustrated by the runs represented in the five panels, *cf*. the Discussion

To complement the information obtained from the UV–Vis measurements, ethyl acetate extracts of sample supernatants at different times of dye removal were analyzed by HPLC. Figure 3 illustrates representative chromatograms of the RB-5 dye and of the degradation and biotic samples. The RB-5 chromatogram contained a peak at a retention time of 11.75 min (Fig. 3a). At different culture times, new peaks (at 254 nm) occurred, which were not present in the biotic culture. The chromatograms contained four main peaks with retention times of 12.75, 15.60, 16.00, and 16.55 min (Fig. 3b–e). Since these peaks were not present in the starting dye chromatogram, they would represent the degradation products of RB-5. After 44 h, the main peak (at 11.75 min) in the dye chromatogram had diminished in height by about 45-fold. The new peak at 12.75 decreased in intensity at 50 h and thereafter decreased no further. That peak was also split, but no further changes were observed during the remainder of the analysis. As the incubation proceeded, peaks appeared at retention times 15.60 and 16.00 min and then changed in intensity until the heights were reversed. The peak that appeared at 16.00 min in the chromatogram corresponded to a metabolite that could be aerobically metabolized by the F20A strain to a less hydrophobic compound because the peak area decreased and shifted to a lower retention time (15.60 min; (Bonakdarpour et al. 2011). The peak area at 16.55 min did not diminish throughout the culture period, suggesting that either that analyte could not be metabolized by the yeast or that the culture conditions were unfavorable for further degradation. The HPLC profiles obtained were consistent with the existence of a dye-biodegradation mechanism, which interpretation is in agreement with previous studies demonstrating the removal of RB-5 by yeasts via degradation (Jafari et al. 2014). Further research should be undertaken to identify the degradation by-products with an aim at understanding the mechanisms of dye removal by this cold-adapted yeast.

This degradation occurred in stages: The coloration of the dark blue cultures, after passing through violet, shifted to pink; and finally, the supernatant color completely disappeared, at the same time reconfirming that the yeast biomass was colorless. RB-5 has sulfate groups in the *ortho* position of azo bond. Although those moieties provide a steric hindrance for dye reduction; through electrostatic induction and effect of resonance structures, electrons are withdrawn from the associated carbon making that site more electrophilic and thus facilitating the nucleophilic attack in the dye reduction. In addition, the RB-5 molecule has an electron-withdrawing group [–SO_2_(CH_2_)_2_SO_3_Na] at the *para* position of each azo bond on the aromatic ring that stabilizes the negative charge to enhance dye reduction (Hsueh et al. 2009). At the *ortho* position to both azo bonds two different functional groups are present, hydroxyl and amino moieties, that give a different reactivity to each azo bond. The presence of these functional groups is a possible explanation for the aforementioned two-step dye-removal mechanism by *L. muscorum* F20A. This finding had also been reported, among others, by Enayatizamir et al. (2011) for a filamentous fungus and Martorell et al. (2016) for a yeast.

### Effect of different parameters on dye removal

**Fig. 4:**
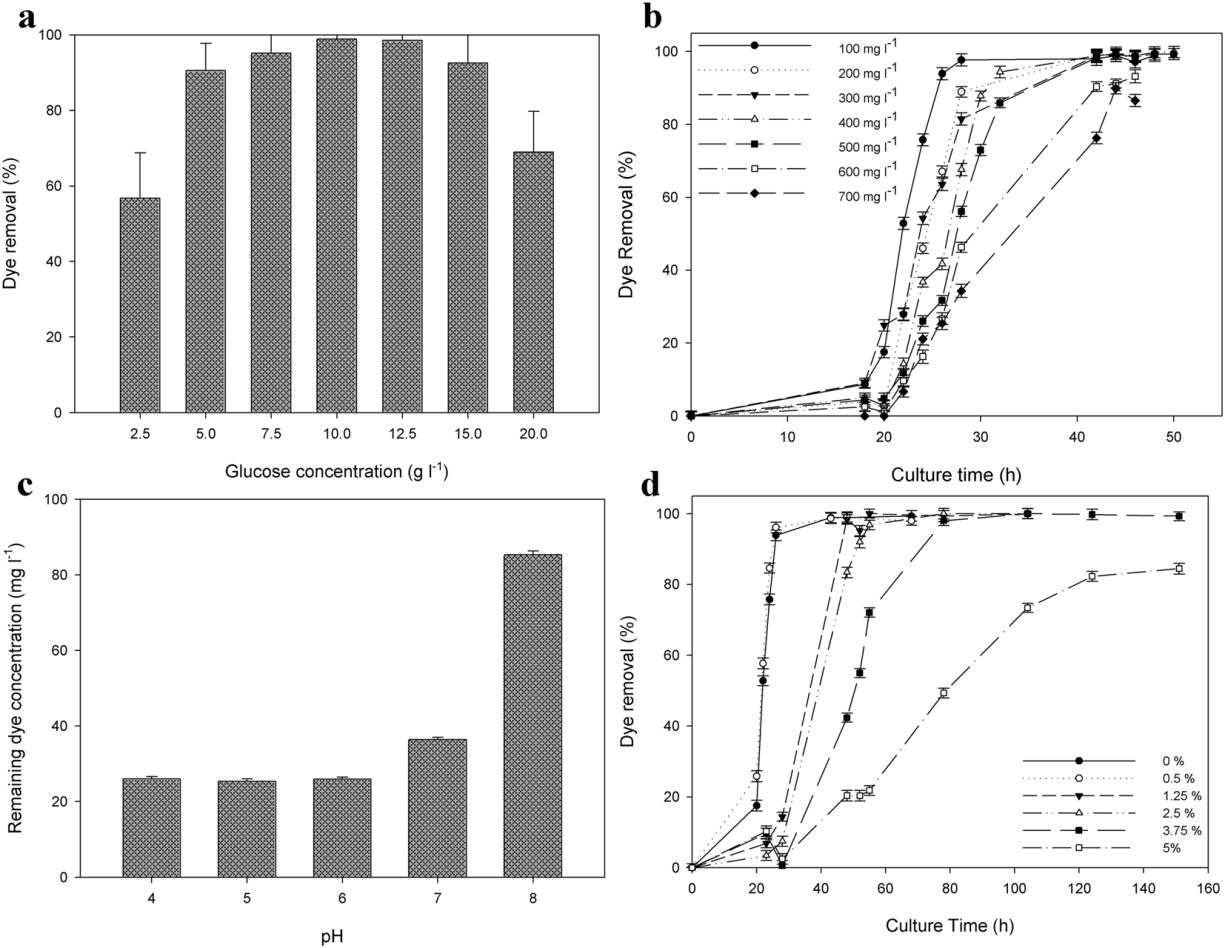
Dye removal by *Leucosporidium muscorum* F20A as a function of different cultivation parameters. Panel a: effect of medium-glucose concentration. In the figure, the percent dye removal after 26 h of cultivation is plotted on the *ordinate* as a function of the initial medium-glucose concentration in g L^–1^ on the *abscissa*. Panel b: effect of Reactive Black-5 concentration. In the figure, the percent dye removal at different dye concentrations is plotted on the *ordinate* as a function of culture time in h on the *abscissa*. Panel c Effect of initial pH. In the figure, the percent dye remaining after cultivation for 26 h is plotted on the *ordinate* as a function of the initial medium pH on the *abscissa*. Panel d Effect of NaCl concentration. In the figure, the percent dye removal is plotted on the *ordinate* as a function of culture time in h on the *abscissa* for different NaCl concentrations, expressed as a percent (w/v). Data are presented as the mean and standard deviation (error bars)

Seven different glucose concentrations were used to determine the optimal concentration for RB-5 removal. Figure 4, panel a illustrates that, after 24 h of culture, only 57 ± 11% of the dye was removed from the culture medium containing 2.5 g L^–1^ of glucose; but when the concentration was increased to 10 g L^–1^, a maximum dye removal (98.9%) was obtained. On the whole, an increase in the glucose concentration effected a higher percent removal; but when the concentration exceeded 15.0 g L^–1^, the removal decreased. As mentioned above, the presence of a cosubstrate is essential for dye degradation. The cosubstrate in this study was glucose, a source of carbon and energy for yeast growth, all of which resulting activity in the microbial metabolism would generate the electrons necessary for the nucleophilic attack on the azo bonds (Ong et al. 2012).

We also studied the influence of the concentration of the dye on its removal, varying that parameter from 100 to 700 mg L^–1^ (Fig. 4d). For concentrations up to 500 mg L^–1^ of RB-5, the NDM medium was used, as stated in Materials and Methods. For the higher concentrations of dye (600 and 700 mg L^–1^), the concentrations of all the medium components were increased by 1.5-fold. In the medium supplemented with 100 mg L^–1^ of RB-5, a complete dye removal was obtained after 26 h of culture. We noted that an increase in the initial dye concentration decreased the removal efficiency over the same time interval (Fig. 4d) although after 45 h of culture the dye removal became equal to or greater than 90% at all the dye concentrations studied. We should also mention that, after 40 h of cultivation, in all instances, the yeast cells remained colorless.

An inhibition of dye removal has been suggested to possibly occur at high dye concentrations that would impede the effectiveness of dye removal. A possible explanation for this inhibition could be due to the toxic effects on microorganisms of the dye and/or certain intermediate compounds (Wang et al. 2009; Tan et al. 2013). In this study, we did not observe a growth inhibition at any dye concentration upon comparing the final biomass reached (g L^–1^) among the dye-supplemented cultures with the biotic control (without RB-5), and thus such a putative inhibition was insignificant. In contrast, we did observe a decrease in dye removal upon increasing the dye concentration, but which for practical purposes would not be a disadvantage because textile effluents generally have a dye concentration of 10 to 200 mg L^–1^ and no higher (Jafari et al. 2013a).

One of the problems in the biologic treatment of textile effluents is their high pH. In this work, we observed that at 26 h of culture the RB-5 removal was performed efficiently up to a pH 7.0 (84 ± 6%; Fig. 4c) although by 48 h of culture *L. muscorum* F20A reached a removal over 96% for all the medium pHs tested. Furthermore, the strain exhibited relatively high removal rates within the pH range of 5.0–6.0. Similar results were reported by other authors for different yeast strains: With *Yarrowia lipolytica* NBRC 1658, the maximum removal rate was obtained at an initial pH of 6.0–7.0 for 50 mg L^–1^ of RB 5 (Aracagӧk and Cihangir 2013); in addition, 200 mg L^–1^ of RB 5 were efficiently removed by *Debaryomyces polymorphus* Y1-0813 within an initial pH range of 5.0–7.0 (Yang et al. 2005) and by *C. palmioleophila* JKS4 at a pH value of 7.0 (82.4%; (Jafari et al. 2013a). Finally, *K. marxianus* IMB3 completely removed Remazol Black-B (up to 100 mg L^–1^) within a range of acidic initial pH values from 3.0 to 5.5 (Meehan et al. 2000).

The initial pH has a major effect on the efficiency of dye removal under all the circumstances studied, with the optimal initial pH value for dye removal by the yeasts being acidic or close to neutrality. A significant decrease in the medium pH was observed at the end of the degradation, which drop may be due to the metabolism of ammonium sulfate by the yeast, but also might be caused by the functional sulfonate groups that were liberated from the dye during the degradation (Yang et al. 2005).

*Leucosporidium muscorum* F20A removed dye at the same rate in the medium with normal salt concentration as in the one supplemented with 0.5% (w/v) NaCl, with 100% of the dye being removed at all the concentrations assayed except for 5%, where an 84.4 ± 1.5% dye removal was nevertheless still obtained after 150 h of culture. As the salt concentration in the medium increased, though, the rate of dye removal decreased.

### Toxicity assessment with seeds from *L. sativa* L

The bioassay with *L. sativa* L seeds can indicate whether or not the germination percentage and the development of the radicle and hypocotyl of the lettuce plant during the first days of growth is affected by the phytotoxicity of pure compounds or complex mixtures (Lyu et al. 2018). The IC_50_ data in Table 4 revealed that, despite the high dye-removal rate by the yeast, that resulting decrease in the dye concentration—implying an increase in the degradation products—caused a 50% inhibition of the root growth, which finding demonstrated an increase in phytotoxicity at all the treatment times analyzed. In agreement with the present results, a previous study by Almeida and Corso (2014) had also pointed to an increase in toxicity after the treatment of an azo dye with microorganisms, with *L. sativa* L. seeds and *Artemia salina* larvae as bioindicators of toxicity. The authors attributed this increase in toxicity to the incomplete dye biodegradation, where the mineralization of the metabolites was not achieved during the process. These metabolites thus exhibited a higher toxicity than the initial dye molecule. Therefore, a total removal of the effluent color does not always constitute a positive outcome, since if the resulting toxicity is greater than the raw effluent, those degradation products should not be discharged in this way into the recipient water bodies.

**Table 4:** Toxicity values of IC50 of *Lactuca sativa* L at different dye treatment times with *Leucosporidium muscorum* F20A. Data are presented as the mean ± DS (*n* = 4)

Test solution	IC_50_ (mg L^–1^)
Dye solution before treatment	30 ± 7
Dye solution after 23 h of treatment	1.7 ± 0.1
Dye solution after 40 h of treatment	0.71 ± 0.08
Dye solution after 72 h of treatment	0.7 ± 0.1
Dye solution after 144 h of treatment	0.26 ± 0.06
Positive control with Zn (II)	142 ± 9

Therefore, the study demonstrated that the yeast *L. muscorum* F20A, though exceedingly effective in effecting RB-5 dye removal, nevertheless increased the phytotoxicity of the treatment area, thus indicating the need to combine that technique with other treatments or else use other microorganisms. For example, a previous report of ours had indicated that *C. sake* 41E—a yeast from the same Antarctic collection—was able to remove and detoxify a simulated effluent containing the RO-16 azo dye (Ruscasso et al. 2021b). One finding that emerges from this work is the necessity of doing toxicologic analyses of treated effluents because an essential and often underestimated aspect of such biodegradations is the toxicity of effluents and the development of that property during wastewater treatment since dye removal results in the formation of colorless but potentially toxic compounds that can increase the toxicity of the wastewater. Furthermore, future research should examine effluent toxicity through simultaneous tests on several microorganisms in order to ensure a better understanding of the impact of pollutants and their biodegradation products on natural environments.

## Conclusion

The results from this study provide a useful background for proposing a new ecologic alternative for treating the wastewater from dye-utilizing industries through the use of cold-adapted yeasts. Most of the yeasts from the present Antarctic collection were able to remove RB-5 dye through different mechanisms, such as bioaccumulation or biodegradation. According to these results, *L. muscorum* F20A may have a potential application in the biotransformation of several textile dyes. Moreover, to the best of our knowledge, this report is the first involving the ability of *L. muscorum* to degrade the azo dye RB-5. Future research should focus on determining the enzymatic mechanism responsible for dye biodegradation and the by-products generated as identified by physicochemical techniques such as tandem HPLC–mass spectrometry.

## Data Availability

All data generated or analyzed during this study are included in this published article.

## Abbreviations

YPD: Yeast extract peptone dextrose
RB-5: Reactive Black 5
RO-16: Reactive Orange 16
RB-19: Reactive Blue 19
AB-74: Acid Blue 74
NDM: Normal decolorization medium
ABTS: 2,2'-Azino-bis(3-ethylbenzothiazoline-6-sulfonic acid) diammonium salt
SYR: Syringaldazine, 3,5-dimethoxy-4-hydroxybenzaldehydazine
OD_600_: Optical density
HLPC: High-performance liquid chromatography
IC_50_: The half maximal inhibitory concentration
*ν*: The average specific removal rate
*η*: The average volumetric-removal rate
